# Nutrition is Associated with Violent and Criminal Behaviors

**DOI:** 10.1007/s13668-025-00668-7

**Published:** 2025-06-03

**Authors:** Esma Asil, Eda Erkmen

**Affiliations:** 1 Faculty of Health Sciences, Department of Nutrition and Dietetics, Ankara University, Fatih Street, Number:197/7, PK:06290, Keçiören, Ankara, Türkiye; 2 Graduate School of Health Sciences, Department of Nutrition and Dietetics, Ankara University, Ankara, Türkiye; 3 Faculty of Health Sciences, Department of Nutrition and Dietetics, Alanya Alaaddin Keykubat University, Antalya, Türkiye

**Keywords:** Nutrition, Behavior, Mood, Crime, Violence

## Abstract

**Purpose of Review:**

Psychological illnesses, mood disorders, anger and violent behaviors, which are increasing at an alarming rate today, not only negatively affect human health but also pose a threat to social life and security. The extant literature indicates that mental illnesses (e.g., depression and anxiety), negative affect (e.g., unhappiness and anger), and antisocial behaviors are associated with an increased likelihood of criminal behavior. Therefore, treating psychological disorders, improving mood and transforming negative behaviors into positive behaviors seems to be a potential strategy for reducing the crime rate and preventing crime. Given the existing literature associating nutrition with mood, behavior, and crime, this narrative review aims to examine the effects of nutrition on violent and criminal behavior.

**Recent Findings:**

Despite the common perception that an unhealthy diet is an effective strategy to improve mood, current research has shown that the opposite is true. The findings showed that healthy eating plays an important role in improving mood, treating psychological disorders and preventing negative behaviors. In addition to the therapeutic effects of a healthy diet, macro- and micronutrient deficiencies have been associated with a range of psychological disorders, including poor mood, violence and criminal behavior.

**Summary:**

A healthy diet with adequate amounts of macro- and micronutrients is essential for mental and physical health, as well as for the prevention and treatment of negative behaviors, and for the well-being, order and security of the individual and society.

## Introduction

The term “crime” is defined as human behavior that expresses the intentional and willful violation of legal values that must be protected in order to maintain social order (i.e., intent) or carelessness (i.e., negligence) towards the rules aimed at protecting these values [1]. Violent and criminal behavior endangers people’s security, which is one of their fundamental rights.

**Fig. 1:**
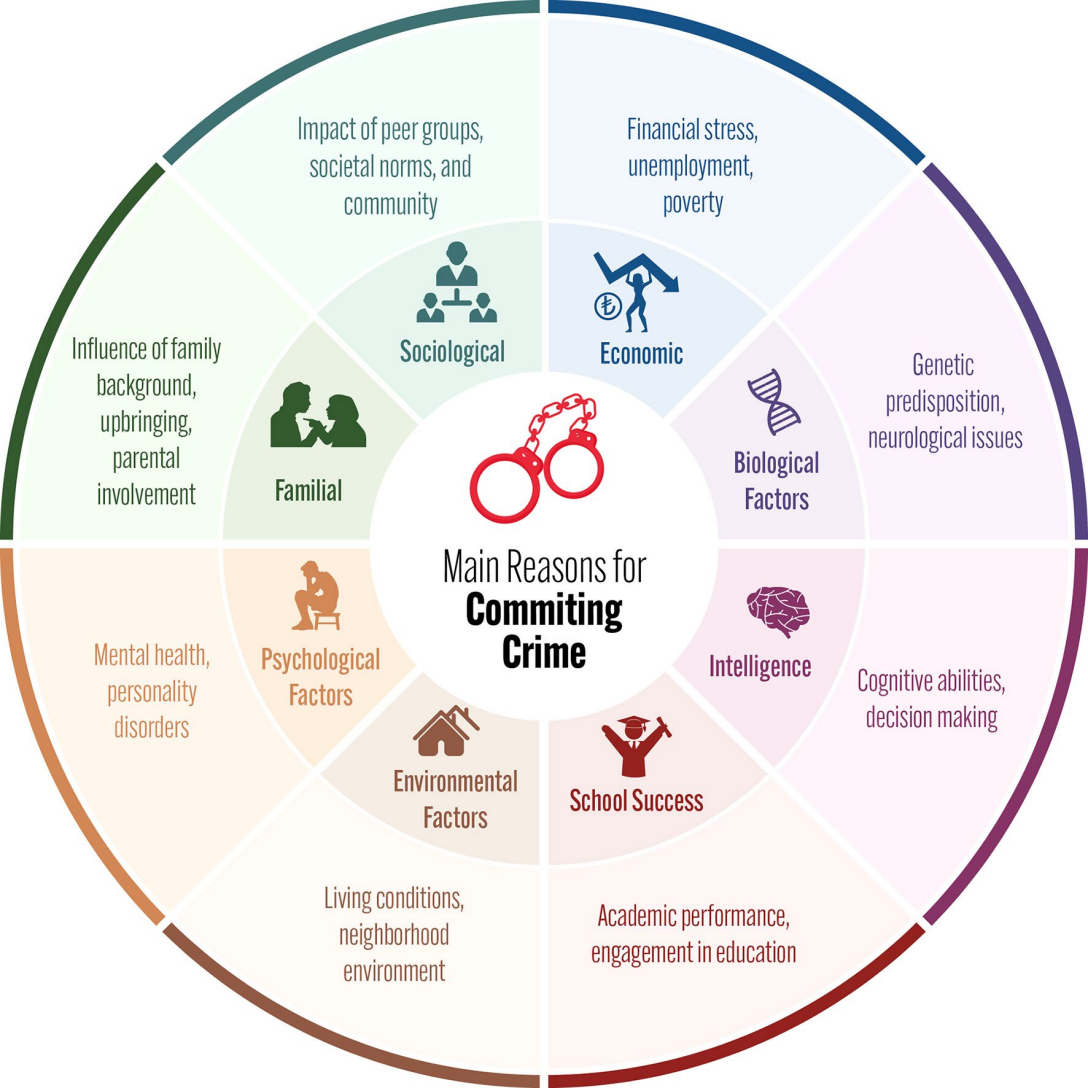
Main reasons for committing crime

Since crime rates are increasing despite existing deterrents, the development of new strategies has gained critical importance. Economic, sociological, familial, familial, environmental, psychological and biological factors, school success and intelligence are among the main reasons that push individuals to commit crimes [2] (Fig. 1).

**Fig. 2:**
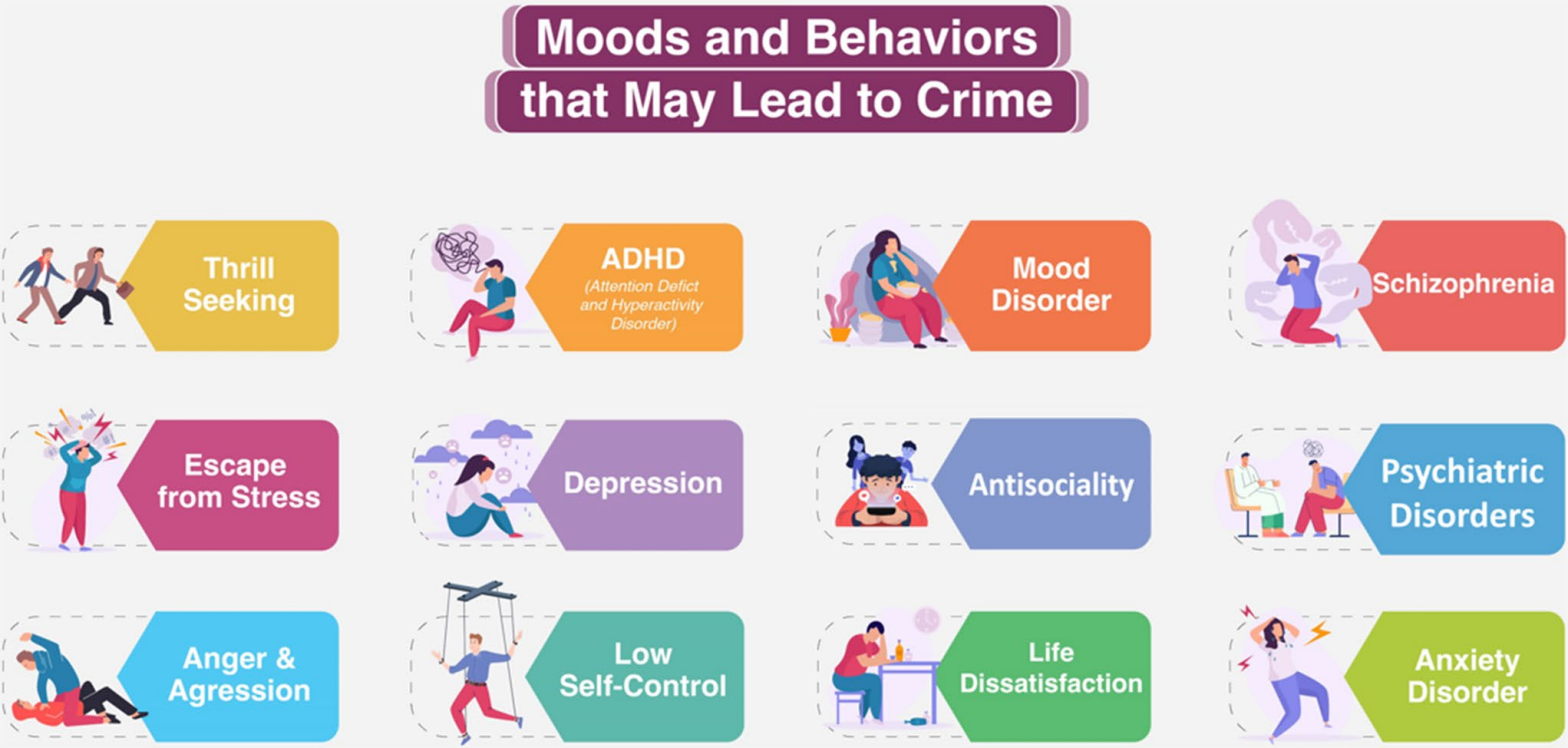
Moods and behaviors that may lead to crime

In different studies, the main moods and behaviors that may lead to offending were found to be psychiatric illnesses, mood disorders and anxiety disorders [3], schizophrenia, stress escape and thrill seeking [4], attention deficit hyperactivity disorder (ADHD) [5], life dissatisfaction and aggression [6], low self-control [7], antisociality, depression and anger [3, 4] (Fig. 2).

While existing literature on the relationship between nutrition and crime has primarily focused on food insecurity, the evidence that nutrition affects a range of human behaviors, including school success, intelligence, psychological factors, biological factors, self-control, mood, mental health, and so forth, suggests that it may also be a contributing factor in violent behavior and crime [8]. It is estimated that nutrition is directly related to both crime and the reasons for committing crime, and that a healthy and proper diet can lead to a decrease in crime rates by eliminating the factors that cause individuals to commit crime. In this review, the effect of nutrition on violent and criminal behaviors and whether it can be used as a strategy to reduce crime rates will be discussed.

In addition to presenting a general picture of the relationship between nutrition and violent and criminal behavior, the present study also provides readers with information on possible mechanisms of the relationship and the role of different nutrients and nutrients in this relationship. The strengths of the study lie in its interdisciplinary approach, combining nutrition and psychology, and in its value as an informative resource for nutritionists/dieticians, psychologists, and psychiatrists regarding the impact of nutrition on mental health. Furthermore, the scarcity of existing literature on the relationship between nutrition and violent or criminal behavior highlights the study’s originality. The findings are expected to encourage further research in this emerging field and may help inform policy development aimed at reducing crime rates through insights from nutritional and psychological sciences.

## Methods

The study is a narrative review. “Scopus”, ‘Web of Science’, ‘PubMed’ and ‘Google Scholar’ databases were used for literature search. The words “nutrition”, “crime”, “violence”, “psychology”, “psychology”, “behavior” and their combinations were used as keywords in the literature search and articles published between 2000 and 2024 were examined. After excluding articles whose full text could not be accessed and articles that were found to be missing important methodological details, 45 studies were included in the review.

### Possible Mechanisms of the Relationship between Nutrition and Crime

There is a complex relationship between nutrition and mood. When people are in a bad mood, they tend to consume more unhealthy foods high in fat, sugar or salt, regardless of their normal eating habits. Increased consumption of unhealthy foods during this period leads to an imbalance of neurotransmitters, which play very important roles in the regulation of mood, causing mood to worsen [9].

### Gut-Brain Axis and Gut Microbiota

The gut-brain axis (GBA) represents the intricate, bidirectional relationship between the gut and the brain. This relationship involves the coordinated communication of the nervous system, immune system, and endocrine system [10]. The fact that the gut microbiota interacts with the central nervous system (CNS) suggests that it has an impact on both brain function and behavior. There are direct and indirect pathways through which the gut microbiota can modulate the gut-brain axis, such as endocrine (cortisol), immune (cytokines) and nervous (vagus and enteric nervous system) pathways [11].

Diet has significant effects on gut microbiota. For instance, *Bacteroides* enterotypes have been linked to diets comprising high levels of fat and protein, whereas *Prevotella* enterotypes have been associated with diets that are high in carbohydrates [12]. Due to the low fiber and high saturated fat (SFA), refined sugar and artificial sweeteners content of the Western-type diet, epithelial permeability may or may not be increased, resulting in a degraded mucus layer and an unhealthy gut microbiome [13]. Probiotics, which also induce changes in the microbiota, are thought to exert their effects on cognitive performance, mood, and behaviors through GBA [14].

A healthy microbiota is characterized by a balanced composition that exerts beneficial effects on the health of the individual. However, disruption of this balance leads to predispose the individual to the development of various diseases [15]. Major depressive disorder in humans is associated with changes in the healthy gut microbiome [16]. In a study on this subject, the gut microbiome and fecal gut microbiota were transferred from depressed humans to rodents and it was observed that depression and depression-like conditions developed in rodents [17].

### Vagus Nerve

The vagus nerve is a major component of the parasympathetic nervous system, which regulates various bodily functions, including mood control, mediating the interaction of the immune system, digestive system and GBA. The microbiota triggers the secretion of proinflammatory cytokines from the innate immune system, and the presence of proinflammatory cytokines affects neuronal physiology through vagal nerve stimulation [18]. It is thought that the effects of microbiota on neuronal physiology may influence behavior and mood. In patients with depression, concentrations of proinflammatory cytokines in plasma and cerebrospinal fluid are elevated relative to normal levels. The inhibitory effect of the vagus nerve on proinflammatory cytokine production is thought to have beneficial impacts on depression [19]. In a multicenter study conducted in Europe, it was found that vagus nerve stimulation therapy was effective in reducing the severity of depression and providing improvement in treatment-resistant depression patients, and this effect gradually increased in the long term [20]. Similar studies have also reported that vagus nerve stimulation is a highly effective method for treating depression [21–23]. The observation that rats administered Bifidobacterium longum to induce intestinal dysbiosis exhibited reduced anxiety levels compared to control subjects, and that subsequent vagotomy (vagus nerve cutting) prevented this effect, lends support to the hypothesis that there is a relationship between microbiota and mental health, and thus crime [24]. Studies show that dietary components such as probiotics and gluten have a significant effect on the activity of the vagus nerve by interacting with the gut microbiota [25, 26]. This is considered to be one of the mechanisms that may prove effective in eliminating the causes of offending through the effect of nutrition on microbiota and mental health.

### Crosstalk Mechanism

One of the important mechanisms involved in GBA interaction is crosstalk. In the human body, undigested carbohydrates such as fiber are fermented into short-chain fatty acids. Short-chain fatty acids trigger the secretion of cholecystokinin (CCK) and glucagon-like peptide-1 (GLP-1) from enteroendocrine cells in the gastrointestinal tract [27]. Endocrinologically active compounds such as CCK and GLP-1 elicit alterations in host physiology and behavior. Microbiota and host interactions of this nature are called cross-talk [28]. Since crosstalk mechanisms are involved in the pathophysiology of anxiety disorders and depression, they are becoming a target in the treatment of these diseases. One of the compounds affecting host behavior through crosstalk is tryptophan (Trp), a serotonin precursor [29].

Biologically active nutrients affect neural processes in brain regions central to mood regulation. Mood disorders and weakening of this neural network are associated with altered serotonin levels [30]. Nutrients containing tryptophan influence serotonin levels in the brain and regulate the nervous system in a manner that is conducive to maintaining positive mood [31]. Tryptophan engages in competition with other large neutral amino acids (LNAA) for a transport molecule that permits entry into the brain. This limits the possibility that nutrients can increase brain tryptophan levels and improve mood. However, high-carbohydrate diets induce insulin, which increases the uptake of other LNAAs into peripheral tissues, accelerating the uptake of tryptophan into the brain and reducing competition [32]. In a study with 32 women in their second postmenstrual week, participants consumed a control drink with a Trp/LNAA ratio of 0.02 and a test drink with a Trp/LNAA ratio of 0.19, 1–4 months apart. At the end of the study, it was found that increasing Trp/LNAA ratios of food improved mood by affecting neural networks [30].

### Immune System

Recent studies show that another factor that plays a role in the pathogenesis of violent behavior is changes in the immune system [33–35]. The results of a study conducted on this subject indicated a positive association between oxidative stress biomarkers and the levels of aggression exhibited by individuals [36]. In another study, it was demonstrated that inflammation may be a contributing factor in the development of antisocial personality disorder, which is characterized by illegal and unethical behaviors, impulsivity, risk-taking, and aggression [37]. In a study in which individuals with a diagnosis of bipolar disorder were divided into two groups according to their offense status, it was found that previous offense was positively associated with inflammatory biomarkers [38]. In a study comparing schizophrenia patients who had and had not committed crimes before, it was found that inflammatory biomarkers were significantly higher in criminal schizophrenia patients, some inflammatory biomarkers were positively correlated with aggression and impulsivity, inflammatory biomarkers differed according to the type of crime, and those who committed murder had significantly higher CRP levels [39]. Many nutrients are being investigated for their anti-inflammatory properties and their use in the treatment of inflammation. Studies have found that malnutrition, Western-type nutrition model, trans fatty acids and SFAs may cause inflammation, whereas Mediterranean diet, DASH diet, polyphenols, fiber, omega-3 and omega-6 fatty acids have anti-inflammatory properties [40].

### The Relationship between Food, Nutrients and Crime

There is a complex, cyclical interaction between nutrition and mood. Choosing the right food plays an important role in improving mood. Different foods and nutrients have different effects on mood [9]. The effect of nutrition on behavior is thought to be due to its important roles in brain function and neurotransmitter production [41]. A summary of various studies investigating the relationship between food and nutrients and the causes of offending is given in Table 1.

**Table 1:** Studies in which nutrients and nutrients are associated with the causes of criminal behavior

Food and nutrients	Mechanism of action	The relationship with crime	Ref.
**Carbohydrate**			
↑ Glycemic index and processed carbohydrates	Repeated and rapid changes in blood glucose	Increased risk of depression and anxiety	[32, 42–46]
	Triggering the inflammatory process	Increased symptoms of depression	
	Lead to the secretion of regulatory hormones against hyperinsulinemia	Anxiety, irritability, behavioral changes	
Chocolate	Psychoactive agents such as anandamide etc.	Antidepressant effect	
	Excessive consumption	Anxiety disorder	
**Protein**			
Tryptophan	Increasing Trp/LNAA ratios of nutrients	Improvement in mood	[30, 31, 47–49]
Low consumption of meat products	Inadequate intake of serotonin precursor tryptophan	Depression, anxiety disorders	
**Fat**			
Omega-3 fatty acids	DHA affects neurotransmitter function and EPA affects neuron function	Decline in anger and anxiety scores	[50–55]
	Affect the number and affinity of neurological receptors	Prevention of aggression	
	Involvement in neuronal structure and function	Reduced levels of aggression	
	Omega-3 fatty acids less than 6% of total erythrocyte fatty acids	Reduced aggression, hostility and attention deficit	
	Enhance neurite outgrowth, regulate neurotransmitter functioning and gene expression	Decrease in behavioral problems	
Cholesterol	Low serotonin levels due to low cholesterol intake	Risk of violence in individuals with trauma	
**Fiber**			
Soluble and insoluble fiber	Synthesizing endocrinologically active molecules such as CCK and GLP-1	Decreased risk of depression	[44]
**Vitamins and minerals**			
Folate deficiency	Decreased effectiveness of antidepressant drugs	Increase in depressive symptoms	[56, 57]
Vitamin B, vitamin C, vitamin D and zinc deficiency	Balancing neurotransmitters that regulate mood	Bad mood and depression	
Iron	Iron has positive effects on appetite and nutritional status	Increase in IQ score	
**Water and other beverages**			
Inadequate water consumption	Renin-angiotensin II-aldosterone system and the effect of arginine on vasopressin	Bad mood, depression, anxiety, irritability	[58–60]
Caffeine	Psychoactive substance	Protective effect against depression	
	Ergogenic effect of caffeine	Reducing morning tension	
**Spices**			
Spicy food and red pepper consumption	Contains capsaicin	Anger, excitement and reward seeking	[61–63]
Capsaicin	Effects on dopamine and serotonin	Social dominance and aggression	
	Effects on dopamine and serotonin	Novelty seeking and adventurous behavior	
**Probiotics and prebiotics**			
Mixture of *Lactobacillus Helveticus* and *Bifidobacterium Longum*	Impact on gut microbiota	Alleviation of psychological distress	[64, 65]
Milk drink containing *Lactobacillus casei*	Impact on gut microbiota	Improvement in mood	
**Nutritional supplements**			
Vitamin-mineral supplements	Effects on cognitive function	Decrease in the number of reported cases in prisoners	[66, 67]
Vitamin, mineral and essential fatty acid supplementation	Effects on cognitive function	Reduction in violence and antisocial behavior	
**Alcohol**			
Excessive alcohol consumption	Loss of consciousness and self-control	Assault, rape and theft crimes	[68, 69]
Risky alcohol consumption	Loss of consciousness and self-control	Assault crime and traffic accidents	
**Mediterranean Diet**			
↑ Fiber, polyphenol, MUFA, PUFA content	Supporting gut microbial taxa that can metabolize nutrients into anti-inflammatory metabolites such as SCFA	A healthy gut microbiome	[13]
**Western dietary pattern**			
↑ SFA, refined sugar, artificial sweetener content	Impaired mucus layer with or without increased epithelial permeability	An unhealthy gut microbiome	[13]

Trp: tryptophan, LNAA: large neutral amino acids, DHA: docosahexaenoic acid, EPA: eicosapentaenoic acid, CCK: cholecystokinin, GLP-1: glucagon-like peptide-1, IQ: intelligence quotient, MUFA: monounsaturated fatty acids, PUFA: polyunsaturated fatty acids, SCFA: short-chain fatty acids, SFA: saturated fatty acids

### Carbohydrates

When the relationship between carbohydrates, which are important components of the diet, and mood is examined, it is estimated that the consumption of foods with high glycemic index or processed carbohydrate content may increase the risk of depression and anxiety by causing repeated and rapid changes in blood glucose [42] and may trigger the inflammatory process and cause the emergence of depression symptoms [32]. Hyperinsulinemia caused by postprandial hyperglycemia due to high glycemic load leads to a dangerous decrease in blood and brain glucose levels. This situation is tried to be balanced with counter-regulatory hormones such as adrenaline, glucagon, cortisol and growth hormone. However, these hormones, which are increased to regulate blood glucose, are also known to cause anxiety, irritability, cognitive impairment, fatigue, and mood and behavioral changes [43]. Gangwish et al. [44] evaluated the relationship between the current nutritional status of postmenopausal women participating in the Women’s Health Initiative study and the risk of developing depression over a 3-year period and showed that depression was associated with high glycemic index, saturated fatty acids (SFA), monounsaturated fatty acids (MUFA) and trans fat intake, low fruit and vegetable consumption, fiber intake and healthy eating index score. In addition, it was determined that higher consumption of added sugar, sucrose and refined grain products increased the risk of depression, while consumption of lactose, fiber, whole grain products, fruits and vegetables decreased the risk of depression. In another study, it was found that consumption of 6.5 servings of vegetables and fruits per day had the highest effect on improving mood. In the same study, consumption of raw vegetables and fruits was associated with fewer depressive symptoms and higher positive mood, life satisfaction and positive functioning, while consumption of carbonated beverages was associated with more depressive symptoms and lower life satisfaction [45].

Chocolate often evokes happy feelings and reduces tension. The antidepressant effect of chocolate is due to its psychoactive chemical content, such as anandamides, which target opioid receptors in the central nervous system [45]. In a study conducted by Moreno-Dominguez et al. [46], excessive chocolate consumption was associated with more anxiety than moderate/low consumption.

### Protein

Protein is another nutrient associated with mood, as tryptophan is a precursor of serotonin. Biologically active nutrients affect neural processes in brain regions central to mood regulation. Mood disorders and weakening in this neural network are associated with altered serotonin levels [30]. Foods containing the serotonin precursor tryptophan (Trp) increase serotonin levels in the brain and modulate the processes in the nervous system that regulate mood in a way that is beneficial for mood [31]. Tryptophan competes with other large neutral amino acids (LNAA) for a transport molecule that allows entry into the brain. This limits the potential for nutrients to increase brain tryptophan levels and regulate mood. However, high-carbohydrate diets induce insulin, which increases the uptake of other LNAAs into peripheral tissues, accelerating the uptake of tryptophan into the brain and reducing competition [32]. This is evidenced by a study conducted by Kroes et al. [30] with women in their second postmenstrual week, which demonstrated that increasing the ratio of tryptophan to other LNAAs in foods improves mood by affecting the neural networks that regulate mood. In studies investigating the relationship between protein and mood; it was found that vegetarians had significantly increased depressive symptoms compared to non-vegetarians [47], less red meat consumption was associated with almost doubling the risk of major depressive and anxiety disorders [48], men with low meat consumption experienced depression more than 2 times and women 1.5 times more than those with normal meat consumption [49], pescetarianism, loco-ovo-vegetarianism and veganism were associated with depressive symptoms [64]. In addition to these results, Messaoudi et al. [64] showed that elimination of any food, regardless of the type of food restricted, and increasing the number of foods eliminated increased depressive symptoms.

### Fat

Oilseeds and seafood are the most important sources of omega-3 fatty acids. Low levels of these fatty acids in the blood are associated with depression and mood disorders [70]. The association of omega-3 fatty acids with mood is due to their effects on the roles of arachidonic acid (AA) in the etiology of depression, suppressing inflammation and markers of immune reactivity, reducing inflammatory eicosanoid production by competing with AA, suppressing proinflammatory cytokine production, and leading to increased expression of brain-derived neurotrophic factor, which is thought to be a natural antidepressant in contrast to the Western diet [71–73]. Omega-3 fatty acids have been associated with good mood and behavior [74], impulse control and major depressive disorder [75], bipolar disorder, schizophrenia and substance abuse [76] in different studies.

### Omega-3 Fatty Acids

It is hypothesized that omega-3 fatty acids may be associated with criminal behavior due to their potential influence on mood and behavior. In a study conducted to examine the relationship between omega-3 fatty acids and anger and anxiety that cause crime; it was found that anger and anxiety scores of participants given omega-3 fatty acids decreased significantly. The current data provide support for the emerging evidence of a link between omega-3 fatty acids and hostility and higher homicide rates in countries with low seafood consumption [50]. A meta-analysis of studies examining the relationship between omega-3 fatty acids and aggression suggests that omega-3 fatty acids may be a cost-effective strategy for preventing aggression in both children and adults [51]. Another study found significant reductions in aggression levels in adult participants who were given omega-3 supplements for 6 weeks [52]. In a study conducted in an Australian prison, it was found that the erythrocyte omega-3 fatty acids of inmates were below 6% of total erythrocyte fatty acids, and that the omega-3 index was negatively correlated with levels of aggressive behavior, especially hostility, as well as attention deficit disorder [53]. In a study with children aged 8–16 years, omega-3 supplementation with fruit juice was given for 6 months and it was found that there was a 42–68% decrease in externalizing and internalizing behavior problems reported by parents, and this effect continued 6 months after the completion of the study [54].

The cycle of violence defines exposure to violence as a child and violent behavior expressed in adulthood. In addition, serum cholesterol level is thought to affect the tendency to violence by causing changes in serotonergic activity. To examine this relationship, in a study conducted with individuals who had recently attempted suicide and who had been exposed to violence as children, it was found that serum cholesterol can change the effect of the cycle of violence and may be associated with the risk of violence in traumatized individuals [55].

### Fiber

Insoluble dietary fiber reaches the colon undigested and is fermented by intestinal microbiota into short-chain fatty acids. These short-chain fatty acids trigger the secretion of cholecystokinin and glucagon-like peptide − 1 from enteroendocrine cells in the gastrointestinal tract [27]. These endocrinologically active compounds cause changes in host physiology and behavior through cross-talk [28]. Since crosstalk mechanisms are involved in the pathophysiology of anxiety disorders and depression, they are becoming targets for treatment [77].

### Vitamins and Minerals

It is a well-established fact that vitamin-mineral deficiencies can cause depression and exacerbate its severity. A study revealed that the majority of individuals experiencing depression had a folate deficiency, which significantly reduced the efficacy of antidepressant medications. Furthermore, the same study highlighted the crucial role of B complex vitamins, vitamin D, vitamin C and zinc in maintaining a positive mood and preventing depression. These vitamins and minerals are essential for balancing and controlling neurotransmitters that regulate mood, and it is vital to consume them with food [56].

A study conducted in a prison in the Netherlands demonstrated that inmates who received nutritional supplements exhibited a significantly lower incidence of aggression and rule violations compared to those who received a placebo [66]. In a groundbreaking study, Gesch et al. [67] demonstrated that providing inmates with vitamin, mineral, and essential fatty acid supplements led to a significant reduction in offending rates. Their findings highlight the crucial role of these supplements in curbing antisocial behaviors, including violence, in correctional settings. This evidence is particularly compelling given that malnourished individuals in society also stand to benefit from these supplements.

### Water and Other Beverages

Although there are individual differences, it is recommended to drink up to 8 cups of water a day and inadequate water consumption is associated with poor mood, depression, confusion, anxiety, fatigue, tension and irritability [58]. Caffeine exerts an effect on mood and behaviour by stimulating the central nervous system and increasing activity, performance and alertness [78]. Tea and coffee, which are widely consumed worldwide, are among the most caffeinated beverages. It has been proposed that individuals who regularly consume coffee may be less susceptible to developing depression [79]. Asil et al. [59] concluded that the consumption of up to four cups of black tea and caffeine intake between 450 and 600 mg may help to prevent depression. Souissi et al. [60] found that caffeine intake can significantly reduce morning tension and improve performance in elite judo athletes. However, excessive caffeine intake may have some negative side effects, including headache, tension, irritability, anxiety, restlessness and stress. Therefore, it is recommended to limit caffeine intake to 300 mg per day [80].

Historically, alcohol has been a significant factor in social participation and the formation of social bonds for a considerable proportion of the population. However, excessive alcohol consumption is a significant risk factor for several adverse conditions, including acute or chronic health problems, involvement in criminal activities, road accidents, and addiction. Alcohol consumption is associated with elevated rates of criminal activity, injury, and substantial economic costs [81]. In a study conducted to examine the relationship between alcohol consumption and crime rates, alcohol consumption was found to be positively associated with assault, rape and theft [68]. A study examining alcohol-attributable crimes and traffic accidents in Australia found that risky alcohol use caused between 1.4 and 7.7 common assaults per 1000 population and between 0.6 and 1.8 serious traffic injuries or fatalities per 1000 population each year [69].

### Spices

Capsaicin (trans-8-methyl-N-vanillyl-6-nonenamide) is the main active ingredient of paprika and is also used as a food additive. It has been used for centuries for medical purposes due to its effects on cholesterol, blood lipids, blood sugar, antioxidative, anti-inflammatory, anti-obesity and analgesic effects [82]. Genetic, psychological, physiological and social factors affect people’s motivation to consume foods containing capsaicin. In some cultures, eating hot peppers is associated with strength, courage and masculinity [83]. In a study, consumption of spicy food and red pepper was associated with anger, excitement and reward seeking [61]. It has been suggested that spice consumption is hormonally (especially testosterone) related to behavior. Capsaicin consumption has been associated with anger and thrill seeking [61], social dominance and aggression [62], novelty seeking and adventurous behavior [63].

### Probiotics and Prebiotics

Administration of probiotics and prebiotics has been shown to reduce stress, improve the integrity of the intestinal barrier and reduce inflammation [84]. In studies, it was found that a probiotic mixture of *Lactobacillus helveticus* and *Bifidobacterium longum* alleviated psychological distress, and participants who were initially depressed had significant improvements in their mood after consuming a milk drink containing *Lactobacillus casei* every day for 3 weeks [64, 65]. In addition to genetic factors and exposure to antibiotics, diet is a potentially modifiable determinant of the diversity and functionality of the gut microbiome throughout life [42]. For example, *Bacteroides* enterotypes are associated with diets high in fat or protein, while *Prevotella* enterotypes are associated with high carbohydrate diets [12]. Western diets are low in fiber and high in SFA, refined sugars and artificial sweeteners, resulting in a degraded mucus layer with or without increased epithelial permeability and an unhealthy gut microbiome. On the other hand, the Mediterranean diet supports a healthy gut microbiome by supporting gut microbial taxa that can metabolize nutrients into anti-inflammatory metabolites such as short-chain fatty acids thanks to its high content of fiber, polyphenols, MUFA, polyunsaturated fatty acids [13].

## Other Components of the Relationship between Nutrition and Crime

The increase in crime rates despite existing deterrents such as criminal sanctions has revealed the need to develop new strategies for crime prevention. Nutrition is an area with a high potential to be a strategy in crime prevention through its effects on mood, behavior, psychology and cognitive functions. Table 2 summarizes some of the studies linking other components of nutrition with the causes of offending.

**Table 2:** Studies associating other components of nutrition with the causes of offending

Component associated with crime	Mechanism	The relationship with crime	Ref.
**Healthy diet, adequate sleep and regular exercise**			
A holistic health strategy regulating exercise, diet and sleep	Effects on serotonin and dopamine	Reducing crime	[32, 85–89]
Skipping breakfast and bad eating habits	Positive self-attitude	Decline in IQ score	
Breakfast high in carbohydrate/protein ratio	Stimulating serotonin release by increasing tryptophan input to the brain	Response in the right parietal lobule of the brain to risky decision making	
Healthy nutrition	Effects of nutrition on mood and cognitive function	Improved attitudes towards oneself, reduced risk of depression	
Low quality nutrition	Neurological and behavioral effects of nutrition	Antisocial behaviors	
Inadequate nutrition	Macro and micronutrient deficiencies	Self-control problems and aggression	
**Intelligence**			
Skipping breakfast, insufficient zinc and iron intake	Low IQ score	Increased risk of offending, antisocial and aggressive behavior	[57, 86]
**Serotonin**			
↓Serotonin	Effects of serotonin on behavior and mood	Antisociality, violence, aggression, impulsivity, depression	[90–93]
Selective serotonin reuptake inhibitors (SSRIs)	The relationship between serotonergic dysfunction and impulsive aggressive behavior	Reduced impulsivity, irritability, anger, aggression and depression	
**Hypoglycemia**			
↓ Blood glucose level	Glucose provides some of the energy needed for self-control	Aggression	[94, 95]
Uncontrolled diabetes	Loss of willpower during an attack of hypoglycemia	Murder, crime	
**Obesity**			
Obesity	Preventing the formation of personal capital and limiting their participation in the labor force	Incentives to commit crime	[96, 97]
Obesity and binge eating disorder	Serotonin deficiency, negative effects of obesity on mental health	Anger and hostility, borderline personality disorder	

### Lifestyle Diet, Sleep and Exercise Habits

Maintaining physical health through healthy eating, adequate sleep and regular exercise positively affects social life and reduces the tendency to commit crime [98]. A study examining data from the Monitoring the Future 2013 study suggested that improvements in adolescents’ health behaviors may also help reduce certain types of crime. A holistic health strategy that regulates exercise, diet and sleep (exercise programs at school and at home, school lunch programs and healthy eating campaigns) not only supports young people’s physical health but also reduces their risk of offending [85].

Aggressive and antisocial behaviors in childhood continue into adulthood and increase the likelihood of future offending. It is known that malnutrition in childhood can affect brain development and lead to behaviors such as ADHD, antisociality and aggression. In a study conducted with twins to examine the relationship between malnutrition and antisocial behavior, it was found that low-quality nutrition in the preschool period led to antisocial behaviors in the primary school period, considering the effect of genes and environment [88]. In another study, self-control problems and aggression against peers were found in moderate and severe malnutrition in early childhood [89].

Nutrition in infancy and childhood has a very important role in growth as well as in the development of intelligence. Studies have shown that low IQ level increases the risk of committing crimes, antisocial and aggressive behavior [57, 86]. Kusumastuti et al. [57] found an increase in the IQ score of children given 7.5 mg/day iron supplementation for three weeks. In another study conducted with children, it was found that malnutrition (7.3 points), skipping breakfast (4.7 points) and poor eating habits (3.2 points) caused a significant decrease in IQ scores of children [86].

### Serum Serotonin Levels

Low serotonin levels are associated with different moods and behaviors, such as antisociality, violence [90], suicidality, aggression [91], impulsivity and depression, which are associated with crime and delinquency [92]. The association between serotonergic dysfunction and aggression has led to the use of selective serotonin reuptake inhibitors (SSRIs) as a means of controlling impulsive violent behavior. When men with at least one prior conviction for a violent crime were given SSRIs for 3 months, reductions in a range of behavioral parameters that may be associated with crime were observed, including impulsivity (35%), irritability (45%), anger (63%), aggression (51%), and depression (62%) [93]. SSRIs are used as antidepressants in the treatment of most psychological disorders. In a study investigating SSRI use and the risk of committing violent crime, it was found that the risk of committing violent crime increased throughout treatment with SSRIs and up to 12 weeks after discontinuation of treatment [99]. Feeding aggressive individuals with serotonin deficiency with high carbohydrate, low protein diets to increase brain serotonin levels may be an effective strategy [100]. Considering the results found in the studies, it is possible to think that SSRI and diet therapy will be more beneficial together.

The central serotonergic system is primarily targeted in neuropsychiatric treatments and is known to modulate decision-making [32]. Studies have shown that serotonin facilitates punishment and behavioral inhibition through serotonergic manipulation [101, 102]. Avoidance of a significant risk, loss or harm is associated with increased serotonin levels. A high carbohydrate/protein ratio in a meal stimulates serotonin secretion by increasing tryptophan entry into the brain. Liu et al., [32] in a study to examine the effect of serotonin on risky decisions, showed that breakfast consumption with a high carbohydrate/protein ratio caused responses in the right parietal lobule of the brain against risky decision-making and eventually the risk taken decreased.

### Blood Glucose Level

Reactive hypoglycemia is a common finding in impulsive offenders who habitually resort to violence. Intimate partner violence affects millions of people worldwide. The need to vent anger is one of the most frequently cited motivations for intimate partner violence among male and female perpetrators. The energy needed for the self-control required to suppress the aggressive impulse is partly provided by glucose. When blood glucose levels are low, people have more difficulty controlling their attention, regulating their emotions and overriding their aggressive impulses. In a study conducted with married couples to examine the relationship between evening blood glucose levels and aggressive impulses and aggressive behaviors, lower blood glucose levels were found to lead to aggression and aggression. These findings remained significant even after controlling for relationship satisfaction [94]. One of the proofs that hypoglycemia can lead to criminal behavior is the Alasdair Padmore case in the United Kingdom in 1993. The defendant, who was diagnosed with type 1 diabetes and using insulin, killed his friend and was proven guilty. However, the defendant was acquitted after it was understood that he was hypoglycemic at the time of the incident. In another similar case, another defendant who had a regular job and no criminal record was sentenced for sexual assault. After being sentenced, it was understood that the defendant had undiagnosed and uncontrolled diabetes. As a result of psychiatric and neurological tests, the patient was found to have poor executive functions, including judgment and planning abilities. These cases strengthen the hypothesis that uncontrolled diabetes in particular may be associated with cognitive impairment among sexual offenders [95].

### Obesity

Obesity is thought to cause situations that may lead individuals to commit crimes. It is known that being obese decreases individuals’ earnings, reduces their participation in the labor force, restricts their professional gains, and prevents their personal capital formation. Since the consequences of obesity may contribute to criminal behavior, a relationship between obesity and crime has been identified. Therefore, public health policies that effectively reduce obesity in society can contribute to both a healthier and safer community [96]. In a study conducted to evaluate the anger levels and anger management of obese individuals, it was found that obese patients with binge eating disorder felt high levels of anger and hostility towards other people and may have borderline personality disorders [97]. It is known that obesity causes low self-esteem and bad attitudes towards oneself [103], which are among the causes of criminal behavior [104]. On the other hand, healthy nutrition improves people’s attitudes towards themselves and decreases depression levels [87]. This is evidenced by the fact that the average body mass index, waist circumference, body weight to height ratio and body fat percentage were significantly higher in female university students who were evaluated as moderately depressed [105]. Erdem et al. [106] found that dietary intervention led to an improvement in the anthropometric measurements of overweight and obese individuals as a result of a 12-week dietary intervention study in which they kept dietary fat intake below 25% of daily energy intake and saturated fat intake below 10% of daily energy intake. This finding suggests that adopting a healthy and low-fat diet model in addition to a healthy diet will result in an improvement in the anthropometric measurements of overweight and obese individuals, resulting in an improvement in their attitudes towards themselves and depression levels. All of these suggest that treatment of obesity and healthy nutrition may reduce people’s motivation to commit crimes.

## Conclusion

In conclusion, the literature review shows that macro- and micronutrient deficiencies and unhealthy eating habits negatively affect mental health and behaviors in addition to physical health. Existing research has shown that nutrition can cause violent and criminal behavior through its effects on mood and behavior. The findings of the study will provide support for nutritionists in developing a nutritional plan for their clients who are diagnosed with a mental illness and who show or tend to show violent or criminal behaviors. For psychologists/psychiatrists, it will provide guidance on the effects of the diet of their clients who show or tend to show violent or criminal behaviors on these behaviors, and by referring their clients to a nutritionist, the negative behaviors of the people can be changed with positive behaviors with a multidisciplinary working method and measures can be taken before causing a new crime. It is important to conduct further research on the relationship between nutrition and crime and whether it can be used as an alternative strategy in crime prevention.

## Data Availability

No datasets were generated or analysed during the current study.

## Abbreviations

AA: Arachidonic Acid
ADHD: Attention Deficit Hyperactivity Disorder
CCK: Cholecystokinin
DHA: Docosahexaenoic Acid
EPA: Eicosapentaenoic Acid
GLP-1: Glucagon-Like Peptide-1
IQ: Intelligence Quotient
LNAA: Large Neutral Amino Acids
MUFA: Monounsaturated Fatty Acids
PUFA: Polyunsaturated Fatty Acids
SCFA: Short-Chain Fatty Acids
SFA: Saturated Fatty Acids
SSRIs: Selective Serotonin Reuptake Inhibitors
Trp: Tryptophan
